# Phenotype variability of infantile-onset multisystem neurologic, endocrine, and pancreatic disease IMNEPD

**DOI:** 10.1186/s13023-016-0433-z

**Published:** 2016-04-29

**Authors:** Sylvie Picker-Minh, Cyril Mignot, Diane Doummar, Mais Hashem, Eissa Faqeih, Patrice Josset, Béatrice Dubern, Fowzan S. Alkuraya, Nadine Kraemer, Angela M. Kaindl

**Affiliations:** Department of Pediatric Neurology, Charité – Universitätsmedizin Berlin, Campus Virchow-Klinikum, Augustenburger Platz 1, 13353 Berlin, Germany; Sozialpädiatrisches Zentrum (SPZ), Center for Chronically Sick Children, Charité – Universitätsmedizin Berlin, Campus Virchow-Klinikum, Augustenburger Platz 1, 13353 Berlin, Germany; Institute of Cell Biology and Neurobiology Charité – Universitätsmedizin Berlin, Campus Mitte, Charitéplatz 1, 10115 Berlin, Germany; Department of Genetics, AP-HP, Armand Trousseau Hospital, Avenue du Dr. Arnold-Netter 26, 75571 Paris, France; Department of Pediatric Neurology, AP-HP, Armand Trousseau Hospital, Avenue du Dr. Arnold-Netter 26, 75571 Paris, France; Department of Anatomy and Pathology, AP-HP, Armand Trousseau Hospital, Avenue du Dr. Arnold-Netter 26, 75571 Paris, France; Department of Pediatric Nutrition and Gastroenterology, AP-HP, Armand Trousseau Hospital, Avenue du Dr. Arnold-Netter 26, 75571 Paris, France; Department of Genetics, King Faisal Specialist Hospital and Research Center, Riyadh, 11211 Saudi Arabia; Department of Pediatric Subspecialties, Children’s Specialist Hospital, King Fahad Medical City, Riyadh, 59046 Saudi Arabia

**Keywords:** Peptidyl-tRNA hydrolase 2, PTRH2, Intellectual deficit, Motor delay, Speech delay, Sensorineural deafness, Hepatosteatosis, Pancreatic insufficiency

## Abstract

Infantile-onset multisystem neurologic, endocrine, and pancreatic disease (IMNEPD) has been recently linked to biallelic mutation of the peptidyl-tRNA hydrolase 2 gene *PTRH2.* Two index patients with IMNEPD in the original report had multiple neurological symptoms such as postnatal microcephaly, intellectual disability, developmental delay, sensorineural deafness, cerebellar atrophy, ataxia, and peripheral neuropathy. In addition, distal muscle weakness and abnormalities of thyroid, pancreas, and liver were found. Here, we report five further IMNEPD patients with a different homozygous *PTRH2* mutation, broaden the phenotypic spectrum of the disease and differentiate common symptoms and interindividual variability in IMNEPD associated with a unique mutation. We thereby hope to better define IMNEPD and promote recognition and diagnosis of this novel disease entity.

**Electronic supplementary material:**

The online version of this article (doi:10.1186/s13023-016-0433-z) contains supplementary material, which is available to authorized users.

## Introduction

The infantile-onset multisystem neurologic, endocrine, and pancreatic disease (IMNEPD; MIM#616263) was recently reported by us as a novel disease entity in two individuals from a consanguineous family of Yazidian-Turkish descent [1]. We further demonstrated the association of a homozygous nonsense mutation in the *PTRH2* gene (MIM*608625) to IMNEPD through functional and molecular data in human and mouse [1]. The two index patients in the original report presented with postnatal microcephaly, moderate intellectual disability, abnormal rhythmic rapid activity on EEG, sensorineural deafness, and delayed speech development. They suffered from distal muscle weakness and delayed motor milestones, and later developed progressive ataxia and progressive cerebellar atrophy. Peripheral demyelinating sensorimotor neuropathy and endocrine abnormalities with affection of the pancreas, thyroid, and liver were furthermore present [1]. Our single-family report was rapidly further supported by a second case without detailed analysis of the disease phenotype [2]. Here, we report five further IMNEPD patients from two consanguineous families with a *PTRH2* missense mutation and discuss their phenotype, thereby illustrating both core and variable features of IMNEPD.

## Material and methods

Informed consent was obtained from the parents of the patients for the molecular genetic analysis, the publication of clinical data, photos, magnetic resonance images (MRI) and studies on fibroblasts. DNA extraction from blood samples and Sanger sequencing was performed using standard protocols. Samples from patients and controls were used in this study with approval from the local ethics committees of the Charité (approval no. EA1/212/08). Quantitative real-time PCR (qPCR) and Western blot were performed with established methods reported previously [1]. Primer sequences are provided in the supplementary data (Additional file 1: Table S3).

## Results

**Fig. 1:**
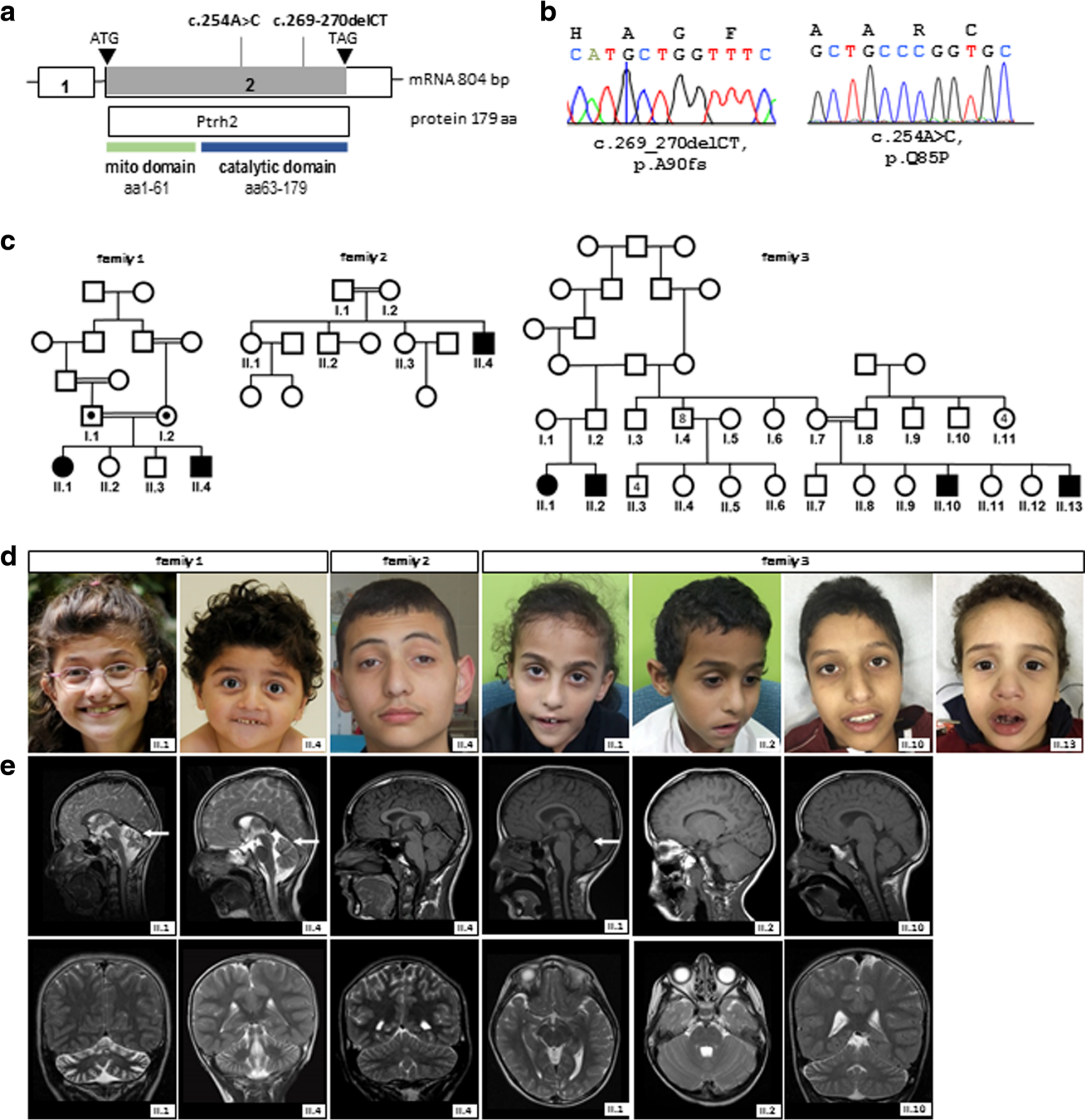
Genotype and phenotype of index patients with IMNEPD. **a** Scheme of *PTRH2* mRNA and protein indicating the site of mutations in exon 2, the only coding exon of *PTRH2.* The mutation c.254A > C was previously reported [2]. **b** Electopherograms depicting the *PTRH2* nonsense mutation of the original IMNEPD family (*left*) and the missense mutation of the further identified IMNEPD families (*right*). **c** Pedigrees of consanguineous families with IMNEPD (left: family 1 previously reported in Hu et al. 2014 [1], middle: family 2, right: family 3). **d** Mild facial dysmorphism of IMNEPD patients with phenotypic variability. Some patients show mild midface hypoplasia, a thin upper lip vermilion, and bilateral ptosis. **e** Cranial MRI of index patients (*top*: sagittal, *below*: coronal plane). Both patients with a *PTRH2* missense mutation (family I, II.2 and II.4) show marked cerebellar atrophy while only one patient with a missense mutation (patient II.1, family 3) was diagnosed with cerebellar atrophy (*white arrows*)

We report five IMNEPD patients from two consanguineous families of Tunisian and Saudi Arabian descent, all with the homozygous missense mutation c.254A > C in exon 2 of the *PTRH2* gene (NM_016077.4; Fig. 1). This mutation causes an amino acid exchange of glutamate to proline (p.Q85P, NP_057161, Fig. 1a) and putatively affects structure, folding, and stability of PTRH2 by altering hydrogen bridge bonds within the protein [2]. In line with this, PTRH2 protein levels were strongly reduced in fibroblasts from patient II.1, family 2, while *PTRH2* mRNA levels were unchanged (Additional file 2: Figure S1). The mutation segregates with the disease phenotype and is heterozygous in the healthy parents of the patients. Patient II.10, family 3, was previously reported, without detailed clinical information, in a large genetic screening study on consanguineous families with developmental delay [2].

**Fig. 2:**
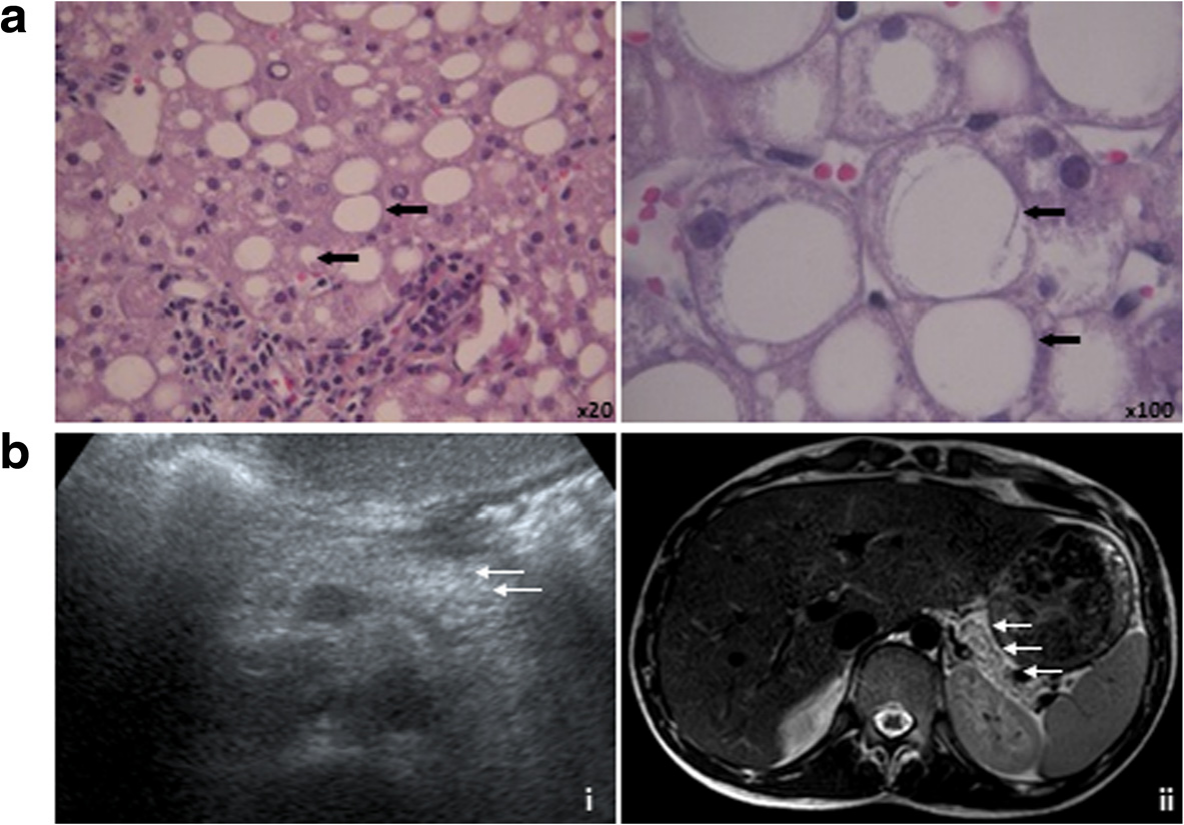
Liver and pancreas affection of IMNEPD patient **a** Paraffin embedded sections of the liver biopsy of index patient II.4, family 2 at x20 magnification (*left*) and x100 magnification (*right*) after haematoxilin safran staining show diffuse micro- and macrosteatosis (*black arrows*). **b** (*i*) Pancreas sonography of index patient II.4, family 2 at age 9 showing hyperechogenicity (*white arrows*). (*ii*) Pancreatic magnetic resonance imaging of patient II.4, family 2 at age 17 showing pancreatic atrophy (*white arrows*)

Neurologic core features of (almost) all patients were intellectual disability, motor delay, severe speech delay, ataxia, and sensorineural hearing loss (Table 1, Additional file 3: Table S1). Furthermore, exocrine pancreatic insufficiency with reduced pancreas elastase levels was detected in almost all patients. It was partly associated with consecutive deficiency of lipophilic vitamins and failure to thrive in the first years of life (Table 1). Insufficiency of the endocrine pancreas function in IMNEPD had already been suggested based on the marginal HbA1c elevation in an index patient of the original IMNEPD family [1] and is now supported by a clinically manifest diabetes mellitus of patient II.4, family 2 (Additional file 4: Table S2). In this patient abdominal sonography at age 9 had shown a hyperechogenic pancreas indicating lipomatosis, and pancreatic atrophy was now diagnosed by magnetic resonance imaging at the age of 17 years (Fig. 2). Hepatomegaly had already been detected in the index patients of the first IMNEPD family [1], but it had remained unclear whether hepatomegaly resulted from fibrosis or steatosis. A liver biopsy performed in patient II.4, family 2, for hepatomegaly and slightly increased transaminase values now revealed hepatic micro- and macrosteatosis (Fig. 1). Slightly increased transaminase values and lactate dehydrogenase activities in further patients could indicate a mild liver (and/or muscle disease) (Additional file 4: Table S2). Despite the identification of the same *PTRH2* mutation in all five patients, phenotypic variability could be observed. For instance, facial palsy, distal muscle weakness, truncal hypotonia, peripheral demyelinating neuropathy, and cerebellar atrophy were only occasionally present (Table 1). Further minor features were skeletal anomalies, especially anomalies of the fingers, and mild facial abnormalities such as exotropia, ptosis, and thin upper lip vermilion.

**Table 1:** Phenotype of index patients with IMNEPD

Ethnic background			Yazidian-Turkish	Yazidian-Turkish	Tunesian	Saudi-Arabian	Saudi-Arabian	Saudi-Arabian	Saudi-Arabian
Mutation			c.269_270delCT	c.269_270delCT	c.254A > C	c.254A > C	c.254A > C	c.254A > C	c.254A > C
Family			01	01	02	03	03	03	03
Pedigree ID (gender)			II.1 (♀)	II.4 (♂)	II.4 (♂)	II.1 (♀)	II.2 (♂)	II.10 (♂)	II.13 (♂))
Age at last assessment (years)			14 3/12	6 8/12	15	7 6/12	5 6/12	13	3
Category	Feature	HPO							
Growth									
Height	Postnatal growth retardation (years at onset)	0001530	+ (11.4)	+ (4)	-	-	-	-	-
Weight	Failure to thrive (years at onset)	0001508	+ (11.4)	+ (4)	-	+ (1.8)	+ (2.2)	-	-
Head and Neck									
Head	Postnatal microcephaly (OFC < P3; years at onset)	0005484	+ (2.5)	+ (0.3)	-	-	-	-	-
	Brachycephaly	0000248	+	+	+	-	-	-	-
Face	Abnormality of the midface	0000309	+	+	(+)	-	-	-	-
	Facial palsy	0010628	+	+	+	+	-	-	-
Ears	Sensorineural hearing impairment	0000399	+	+	+	+	+	+	+
Eyes	Hypertelorism	0000316	+	+	-	-	-	-	-
	Exotropia	0000577	+	+	-	+	-	-	+
Mouth	Thin upper lip vermilion	0000219	+	+	-	+	+	-	-
Abdomen									
Liver	Hepatomegaly	0002240	-	+	+	-	-	-	-
	Abnormal liver parenchyma morphology (on ultrasound)	0030146	+	+	+	-	-	-	-
Pancreas	Exocrine pancreatic insufficiency	0001738	+	+	+	NA	NA	+	+
	Hyperechogenic pancreas	0006276	+	-	+	-	-	-	-
	Pancreatic atrophy (on MRI)	0100800	-	-	+	-	-	-	-
Genitourinary									
External genitalia	Shawl scrotum	0000049	-	+	NA	NA	-	-	-
Skeletal									
Pelvis	Congenital hip dislocation	0001374	+	+	-	-	-	-	-
Hands	Proximal placement of thumb	0009623	+	+	+	+	+	-	-
	Long fingers	0100807	+	+	+	+	+	-	-
	Ulnar deviation of the 2nd and 3rd finger	0009464,0009463	+	-	-	-	-	-	-
Feet	Abnormality of the hallux	0001844	+	-	-	-	-	-	-
	Talipes equinovalgus, incipient	0001772	+	-	+	-	-	-	-
	Achilles tendon contracture	0001771	+	-	-	+	+	+	-
Neurologic									
Central nervous system	Neonatal hypotonia	0001319	+	+	+	-	-	-	-
	Motor delay	0001270	+	+	+	+	+	+	+
	Distal muscle weakness	0002460	+	+	+	+	+	-	-
	Intellectual disability, moderate (IQ 39–70)	0002342	+ (48)	+ (39)	NA	+ (65–70)	+ (55–65)	+ (57)	+
	Dysmetria	0001310	+		NA	+	+	-	-
	Ataxia	0001251	+	+	+	+	+	+	-
	Cerebellar hypoplasia, progressive	0100307	+	+	-	-	+	-	-
	EEG abnormality: alpha-beta-waves even in sleep	0002353	+	+	-	NA	NA	NA	NA
Peripheral nervous system	Demyelinating sensorimotor neuropathy	0003431,0003448	+	+	+	NA	NA	NA	NA
Muscle	Skeletal muscle fibrosis (on ultrasound)	-	+	+	NA	NA	NA	NA	NA
Endocrine features									
	Hypothyroidism	0000821	+	+	-	-	-	-	-
	Diabetes mellitus	0000819	(+)	(+)	+	-	-	-	-
Prenatal manifestations									
Movement	Decreased fetal movement	0001558	+	-	-	-	-	-	-

Abbreviations: *NA* not available, + present, - not present, (+) present, mild

## Discussion

Ptrh2 is an evolutionarily well conserved mitochondrial protein, which prevents the accumulation of dissociated peptidyl-tRNAs and thus an inhibition of protein synthesis [3]. As part of an integrin signaling complex, Ptrh2 regulates the fine balance between cell survival and apoptosis; it also has a role in cell size control [1, 4–7]. We recently highlighted the role of PTRH2 for human development by linking a homozygous *PTRH2* gene nonsense mutation (c.269_270delCT, p.A90fs) to the disease infantile multisystem neurologic, endocrine, and pancreatic disease (IMNEPD) [1]. Since the original description, we have identified five further patients of three consanguineous families of Tunisian and Saudi Arabian descent with an IMNEPD phenotype and a homozygous missense mutation of *PTRH2* (c.254A > C, p.Q85P; Fig. 1). The mutation causes a strong downregulation and a predicted dysfunction of the PTRH2 protein (Additional file 2: Figure S1) [2]. Comparison of the phenotype of the original and the newly identified families illustrates both core features and phenotypic variability of this novel disease entity (Table 1). The core phenotype, mutual to almost all affected patients, comprises intellectual disability, motor and severe speech delay, ataxia, sensorineural hearing loss, and pancreatic insufficiency (Table 1, Additional file 3: Table S1). Progressive cerebellar atrophy and ataxia imposed as key features of IMNEPD in the index family with a homozygous nonsense mutation of *PTRH2*. This was further underlined given the phenotype of mutant *Ptrh2* mice with microcephaly and severe cerebellar atrophy [1]. However, in the light of *PTRH2* missense mutations – presumably with higher residual PTRH2 levels - progressive cerebellar atrophy was present in only one patient. The lack of cerebellar atrophy in some patients with a homozygous *PTRH2* missense mutation and the lack of microcephaly in all patients with a homozygous *PTRH2* missense mutation may be attributed to interindividual variability and/or correlate with the quantity or residual function of PTRH2. Ataxia was present in almost all IMNEPD patients, also in those without cerebellar hypotrophy/atrophy, suggesting that ataxia may not be attributed exclusively to progressive cerebellar atrophy, but could also result from demyelinating peripheral neuropathy detected in several patients (Table 1). Apart from variations in the neurologic phenotype, both patients with the nonsense mutation had hypothyroidism, while thyroxine values in all patients with the missense mutation were normal. However, two patients with a missense mutation presented with elevated thyroxine stimulating hormones (TSH), likely indicating latent thyroid insufficiency. Hepatomegaly and/or abnormal liver parenchyma morphology on ultrasound were present in both patients with a nonsense mutation and one patient with a missense mutation of *PTRH2*. We had speculated before that hepatomegaly and abnormal liver echogenicity were due to fibrosis or steatosis, and we can now demonstrate diffuse and extensive micro- and macrosteatosis in a liver biopsy specimen of patient II.4, family 2. Pancreatic insufficiency is a feature present in most IMNEPD patients: exocrine insufficiency was identified in both patients with a nonsense mutation and four patients with a missense mutation; signs of endocrine insufficiency were found in both patients with the nonsense mutation (borderline HbA1c elevation) and in one patient with the missense mutation (insulin-dependent diabetes mellitus). In the latter pancreatic steatosis and atrophy were diagnosed (Fig. 2). Substitution of lipophilic vitamins in the index patients of the original IMNEPD family markedly improved growth of the patients [1]. The main differential diagnosis of IMNEPD is the syndrome short stature, microcephaly, and endocrine dysfunction (SSMED, MIM#616541), which similarly comprises microcephaly, ataxia, polyneuropathy, endocrine dysfunction, and sporadically cerebellar atrophy. Short stature and microcephaly are already present at birth in patients with SSMED, while they develop postnataly in IMNEPD. Also, patients with SSMED may additionally have ophthalmological and cardiac abnormalities, a thin corpus callosum, and wide cerebral ventricles. Further differential diagnoses include Pearson marrow-pancreas syndrome (MIM#557000), Cockayne syndrome (MIM#216400, MIM#133540), Johanson-Blizzard syndrome (MIM#243800) and metabolic acidosis, encephalopathy, lactate acidosis, and stroke (MELAS, MIM#540000), all depicting a variable combination of sensorineural deafness, ataxia, endocrine abnormalities, pancreas, and/or liver affection. These syndromes can be distinguished from IMNEPD by the additional presence of facial dysmorphism or urogenital defects (Johanson-Blizzard syndrome), ophthalmological, cardiac, or splenic involvement (Pearson marrow-pancreas syndrome, Cockayne syndrome, MELAS), photosensitivity/dry skin (Cockayne syndrome), blood count or bone marrow abnormalities (Pearson marrow-pancreas syndrome, Johanson-Blizzard syndrome), metabolic acidosis (Pearson marrow-pancreas syndrome, MELAS), or encephalopathy (MELAS). With this second report of IMNEPD we are still at the beginning of understanding the genotype-phenotype-correlation and interindividual phenotype variability of the disease. Since IMNEPD affects many organ systems, raising awareness for this disease entity among (pediatric) endocrinologists, gastroenterologists, diabetologists, and neurologists will likely propagate recognition and diagnosis of IMNEPD and, ultimately, improve treatment of affected patients.

## Ethics, consent and publication/consent to publish

Samples from patients and controls were used in this study with approval from the local ethics committees of the Charité (approval no. EA1/212/08). Written informed consent was obtained from the patients’ legal guardian for publication of this case report and any accompanying images.

## Additional files

Additional file 1: Table S3. Primer sequences. (DOCX 14 kb) Additional file 2: Figure S1. PTRH2 protein and mRNA levels in IMNEPD patients with a *PTRH2* missense mutation c.254A > C (p.Q85P). (TIF 26 kb) Additional file 3: Table S1. Development of index patients in the first two years of life. (DOCX 24 kb) Additional file 4: Table S2. Selected laboratory blood values of index patients. (DOC 72 kb)

## Abbreviations

IMNEPD: infantile-onset multisystem neurologic, endocrine, and pancreatic disease
*PTRH2*: peptidyl-tRNA hydrolase 2 gene
MRI: magnetic resonance image

## References

[1] Hu H, Matter ML, Issa-Jahns L (2014). Mutations in PTRH2 cause novel infantile-onset multisystem disease with intellectual disability, microcephaly, progressive ataxia, and muscle weakness. Ann Clin Transl Neurol.

[2] Alazami AM, Patel N, Shamseldin HE (2015). Accelerating novel candidate gene discovery in neurogenetic disorders via whole-exome sequencing of prescreened multiplex consanguineous families. Cell Rep.

[3] Menninger JR (1976). Peptidyl transfer RNA dissociates during protein synthesis from ribosomes of Escherichia coli. J Biol Chem.

[4] Griffiths GS, Grundl M, Leychenko A (2011). Bit-1 mediates integrin-dependent cell survival through activation of the NFkappaB pathway. J Biol Chem.

[5] De Pereda JM, Waas WF, Jan Y, Ruoslahti E, Schimmel P, Pascual J (2004). Crystal structure of a human peptidyl-tRNA hydrolase reveals a new fold and suggests basis for a bifunctional activity. J Biol Chem.

[6] Heurgue-Hamard V, Mora L, Guarneros G, Buckingham RH (1996). The growth defect in Escherichia coli deficient in peptidyl-tRNA hydrolase is due to starvation for Lys-tRNA(Lys). EMBO J.

[7] Jan Y, Matter M, Pai JT (2004). A mitochondrial protein, Bit1, mediates apoptosis regulated by integrins and Groucho/TLE corepressors. Cell.

