# Supplementary material for: Phenotype variability of infantile-onset multisystem neurologic, endocrine, and pancreatic disease IMNEPD
Source: Orphanet J Rare Dis. 2016 Apr 29;11:52. doi: 10.1186/s13023-016-0433-z (PMC4850685; doi:10.1186/s13023-016-0433-z)
Supplement: Additional file 1: Table S3. — Primer sequences. (DOCX 14 kb) [file 13023_2016_433_MOESM1_ESM.docx]

**Supplementary Table 3. Primer sequences**

| **primer** | **Sequence (5’-3’)** |
| --- | --- |
| PTRH2_ex1_for | TGTGGCAAGAGGAAGAATGA |
| PTRH2_ex1_rev | CTGTAAGGAGCCAACCAACC |
| PTRH2_ex2a_for | GGATGACATTCCTTTGTTTTATGA |
| PTRH2_ex2a_rev | CATGCTGGGCAATTATCTTG |
| PTRH2_ex2b_for | TCTCCTGTTTCTGGTGAGATGA |
| PTRH2_ex2b_rev | CTCCAGTGCAATCAGCTCCT |
| PTRH2_ex2c_for | TTTAAGGAACAGCTGCATAGGA |
| PTRH2_ex2c_rev | CGAGGCTCTTTTATAATAGTGTTGAA |
| PTRH2_ex3a_for | TTTTTCTGGATGACCATTAAGTGTA |
| PTRH2_ex3a_rev | TTCATCAGGAGCTTTGACCA |
| PTRH2_ex3_UTR_for | TCCTGAAATGCTCAAACAATG |
