# Supplementary figures and images for: Phenotype variability of infantile-onset multisystem neurologic, endocrine, and pancreatic disease IMNEPD
Source: Orphanet J Rare Dis. 2016 Apr 29;11:52. doi: 10.1186/s13023-016-0433-z (PMC4850685; doi:10.1186/s13023-016-0433-z)

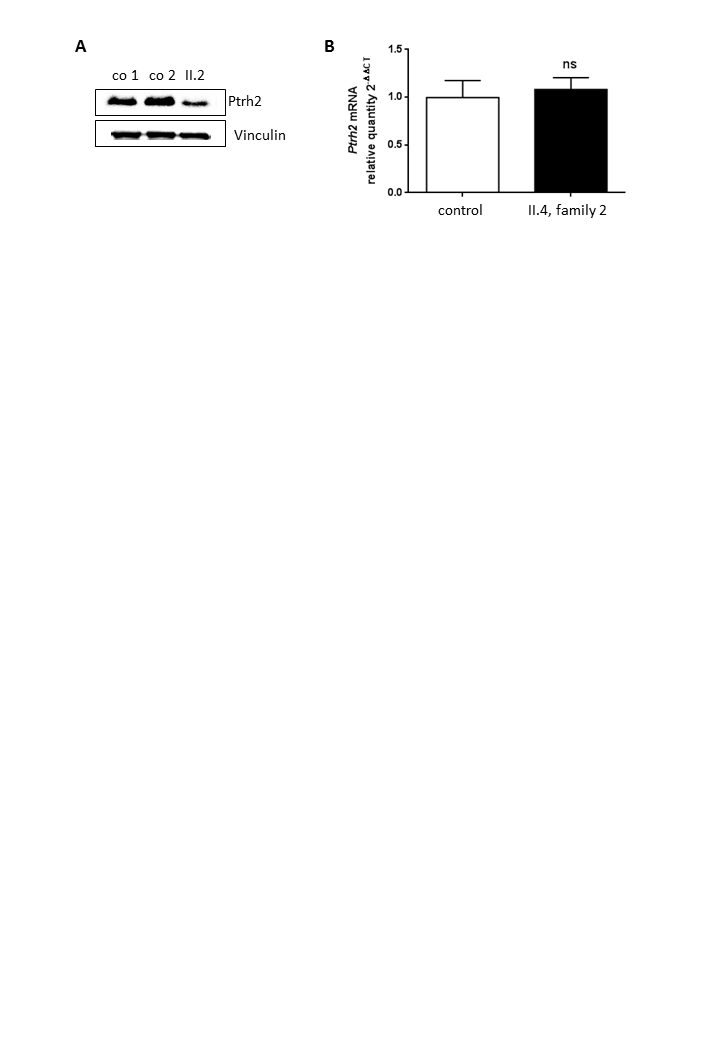

Supplement: Additional file 2: Figure S1. — PTRH2 protein and mRNA levels in IMNEPD patients with a PTRH2 missense mutation c.254A > C (p.Q85P). (TIF 26 kb) [file 13023_2016_433_MOESM2_ESM.tif]
