# Supplementary material for: Phenotype variability of infantile-onset multisystem neurologic, endocrine, and pancreatic disease IMNEPD
Source: Orphanet J Rare Dis. 2016 Apr 29;11:52. doi: 10.1186/s13023-016-0433-z (PMC4850685; doi:10.1186/s13023-016-0433-z)
Supplement: Additional file 3: Table S1. — Development of index patients in the first two years of life. (DOCX 24 kb) [file 13023_2016_433_MOESM3_ESM.docx]

| **Mutation** | | c.269_ 270delCT | c.269_ 270delCT | c.254A>C | c.254A>C | c.254A>C | c.254A>C | c.254A>C |
| --- | --- | --- | --- | --- | --- | --- | --- | --- |
| **Family ID** | | 01 | 01 | 02 | 03 | 03 | 03 | 03 |
| **Pedigree ID** | | II.1 | II.4 | II.4 | II.1 | II.2 | II.10 | II.13 |
| **Features** | **Norms** [months] |  |  |  |  |  |  |  |
| Lifts head for seconds while prone  prone | 2 | 18 | 5 | NA | 5 | 2 | 8 | 4 |
| Rolls from prone to supine | 5-6 | 12 | 8 | NA | 11 | 6 | 12 | 6 |
| Transfers object from hand to hand | 5-6 | 27 | ND | NA | 23 | 24 | NA | NA |
| Babbles | 5-6 | 13 | 18 | NA | 24 | 36 | 36 | 24 |
| Sits with support | 5-6 | 24 | 9 | NA | 16 | 15 | NA | NA |
| Says „da-da“, „ba-ba“ | 7 - 8 | 48 | 24 | 9 | 19 | 60 | NA | NA |
| Sits well without support | 9-10 | 18 | 12 | 9 | 15 | 17 | 17 | 12 |
| Pulls self to sit | 9-10 | 36 | ND | NA | 18 | 20 | NA | NA |
| Waves „bye – bye“ | 9-10 | 36 | 24 | NA | 84 | 60 | 36 | 24 |
| Walks with assistance | 11-12 | 21 | 11 | 17 | 18 | 20 | 24 | 17 |
| Uses two to four words with meaning | 11-12 | 60 | 36 | 48 | 72 | 42 | NA | NA |
| Creeps well | 11-12 | 21 | ND | NA | 22 | 24 | NA | NA |
| Walks by self | 13-15 | 36 | 18 | 22 | 20 | 26 | NA | NA |
| Two- or three-word sentences sentences | 24 | 60 | no (46) | 36 | 84 | 42 | NA | NA |
|  |  |  |  |  |  |  |  |  |

**Supplementary Table 1. Development of index patients in first two years of life**

Abbreviations: NA (not available); ND (not detected)
