# Supplementary material for: Phenotype variability of infantile-onset multisystem neurologic, endocrine, and pancreatic disease IMNEPD
Source: Orphanet J Rare Dis. 2016 Apr 29;11:52. doi: 10.1186/s13023-016-0433-z (PMC4850685; doi:10.1186/s13023-016-0433-z)
Supplement: Additional file 4: Table S2. — Selected laboratory blood values of index patients. (DOC 72 kb) [file 13023_2016_433_MOESM4_ESM.doc]

**Supplementary Table 2. Selected laboratory blood values of index patients**

| **Mutation** |  | c.269_270delCT | c.269_270delCT | c.254A>C | c.254A>C | c.254A>C | c.254A>C | c.254A>C |
| --- | --- | --- | --- | --- | --- | --- | --- | --- |
| **Family ID** |  | 01 | 01 | 02 | 03 | 03 | 03 | 03 |
| **Pedigree ID (♀/♂)** |  | II.1 (♀) | II.4 (♂) | II.4 (♂) | II.1 (♀) | II.2 (♂) | II.10 (♂) | II.13 (♂) |
| **Age at presentation** |  | 14 3/12 | 6 8/12 | 14 | 7 6/12 | 5 6/12 | 13 | 3 |
| **Value** | **Unit** |  |  |  |  |  |  |  |
| Blood count |  | normal | normal | normal | normal | normal | normal | normal |
| CK | U/L | 324 (<210) | 164 (< 128) | 189 (<210) | 52 (<128) | 63 (<210) | 104 (<210) | 91 (<210) |
| Bilirubin, total | μmol/L | 6,84 (<17) | 10,3 (<17) | <17 (<17) | 2 (<17) | 2 (<17) | 3 (<17) | 2 (<17) |
| ALT | U/L | 46 (<31) | 50 (<41) | 94 (<41) | 15 (<31) | 25 (<41) | 41 (<41) | 68 (<41) |
| AST | U/L | 43 (<46) | 60 (<50) | 55 (<46) | 25 (<50) | 47 (<53) | 30 (<46) | 58 (<71) |
| LDH | U/L | 274 (120-300) | 341 (120-300) | NA | 308 (120-300) | 264 (120-300) | 250 (120-300) | 258 (120-300) |
| Lipase | U/L | 5 (<60) | 4 (<60) | 8 (<60) | 7 (<60) | 9 (<60) | NA | NA |
| AP | U/L | 311 (<187) | 273 (<300) | 9 (<390) | 260 (<300) | 210 (<300) | 311 (<390) | 273 (<269) |
| HbA1c | % | 6.5 (<6) | 5 (<6) | 11.5 (<6) | 5.9 (<6) | 6.2 (<6) | 5.5 (<6) | NA |
| TPZ-INR |  | 1.28 (0,9-1,25) | 1.34 (0,9-1,25) | 1.0 (0,9-1,25) | 1.1 (0,9-1,25) | 0.9 (0,9-1,25) | NA | NA |
| aPTT | s | 41.1 (26-40) | 38.8 (26-40) | NA | 32.2 (26-40) | 32.1 (26-40) | NA | NA |
| TSH | mU/L | 3.97* (0.36-5.8) | 4* (0.37-6) | 2.06 (0.37-6) | 0.60 (0.35-4.94) | 1.42 (0.35-4.94) | 10.6 (0.37-6) | 17.6 (0.55-7.1) |
| fT4 | pmol/L | 13.8* (8.7-14.5) | 13.7* (9.5-14.7) | 9.12 (9.1-15.3) | 20.2 (12-22) | 18.8 (12-22) | 13 (10-26) | 13.7 (10-26) |
| 25-OH-vitamine D | nmol/L | 15.3 (50-150) | 17.4 (50-150) | 10 (50-150) | 58.5 (75-250) | 50.5 (75-250) | 65 (75-250) | 83 (75-250) |
| Vitamin A | μmol/L | 0.7 (0.9-2.5) | 0.41 (0.7-1.5) | 0.67* (0.9-2.5) | 0.7 (0.9-1.7) | 1.2 (0.7-1.5) | 1 (0.9-2.5) | 0,7 (0.7-1.5) |
| Vitamin B12 | pmol/l | 100.4 (191-663) | 121.8 (191-663) | 204 (191-663) | 345 (191-663) | 379 (191-663) | 206 (191-663) | 237 (191-663) |
| Vitamin E | μmol/L | 12.3 (13-24) | 2.6 (7-21) | 4* (13-24) | 13.4 (13-24) | 23.9 (13-24) | 11.6 (13-24) | 14.2 (13-24) |
| Pancreas elastase in stool | μg/g | <15 (>200) | <15 (>200) | <50 (>200) | NA | 57 (>200) | 9 (>200) | 6 (>200) |

Pathologic values are highlighted in grey. Normal, age-dependent ranges of laboratory values are given in brackets.

Abbreviations: *(under external substitution); NA (not available)
